# Advanced Characterization of 2D Materials Using SLEEM/ToF

**DOI:** 10.3390/nano16090501

**Published:** 2026-04-22

**Authors:** Veronika Pizúrová, Jakub Piňos, Lukáš Průcha, Ivo Konvalina, Klára Beranová, Oleksandr Romanyuk, Luca Bertolla, Ilona Müllerová, Eliška Materna Mikmeková

**Affiliations:** 1 Institute of Scientific Instruments of the CAS, Královopolská 147, 612 64 Brno, Czech Republic; pizurova@isibrno.cz (V.P.); ilona.mullerova@isibrno.cz (I.M.);; 2 Institute of Physics of the CAS, Cukrovarnická 10/112, 162 00 Prague, Czech Republic; 3 Institute of Physics of Materials of the CAS, Žižkova 513/22, 616 00 Brno, Czech Republic; bertolla@ipm.cz

**Keywords:** 2D materials, graphene, h-BN, MoS_2_, Ti_3_C_2_, SLEEM, ToF, EELS, Raman spectroscopy, XPS, IMFP

## Abstract

Two-dimensional (2D) materials exhibit electronic and collective excitation properties that are highly sensitive to surface chemistry and thickness, requiring surface-sensitive characterization at low electron energies. Here, we investigate graphene, hexagonal boron nitride (h-BN), molybdenum disulfide (MoS_2_), and titanium carbide (Ti_3_C_2_) MXene using an advanced home-built scanning low-energy electron microscopy system combined with time-of-flight electron spectroscopy (SLEEM/ToF). The system uniquely records electron energy-loss spectra (EELS) from transmitted electrons rather than from the reflected electrons used in conventional SLEEM. Compared with high-energy EELS, our low-energy ToF-EELS approach offers enhanced surface sensitivity and reduced beam-induced damage, enabling direct probing of π and π + σ plasmon excitations. Additionally, complementary techniques, including scanning transmission electron microscopy (STEM), Raman spectroscopy, and X-ray photoelectron spectroscopy (XPS), were employed to characterize structural and chemical properties. EELS were acquired for all investigated 2D materials at electron landing energies of 500–1500 eV, and in the 5–50 eV range for selected materials, including graphene and MoS_2_. Analysis of these spectra enabled determination of the average plasmon positions across the measured energy range for all studied materials. Furthermore, a quantitative determination of the inelastic mean free path (IMFP) was achieved for graphene in the 10–50 eV range, yielding a value of 1.9 ± 0.2 nm. These results demonstrate the potential of SLEEM–ToF for surface-sensitive analysis of 2D materials.

## 1. Introduction

Many interesting properties emerge when at least one dimension of a material is reduced to a sub-nanometer size and forms a two-dimensional material (2D). In such systems, the motion of particles, such as electrons, is constrained, leading to entirely new physical behavior and properties of the material absent in its bulk counterparts. These extraordinary electrical, mechanical, and optical properties have opened up exciting possibilities for next-generation electronics, optoelectronics, energy storage, sensing, medicine, and quantum computing. This revolution in material science and condensed matter physics began with the discovery of graphene, a single-atom layer of carbon atoms arranged in a hexagonal lattice [1]. Graphene exhibits exceptional properties, including high thermal conductivity (up to 5300 W/mK) at room temperature [2], high electrical conductivity up to 1.46 MS/m [3], low optical absorption of 2.3% [4], high electron mobility of 10,000 cm^2^/(V·s) [5], and high Young modulus of 1100 GPa [6].

While graphene is a remarkable semimetal, creating functional electronic devices requires a full spectrum of materials: conductors, semiconductors, and insulators. Thus, it is desired to search for 2D candidates that fulfill these distinct roles. The exploration has already unveiled diverse families of atomically thin materials with a remarkable range of tunable properties, such as transition metal dichalcogenides (TMDs: WS_2_, MoS_2_, etc.) [7], MXenes (Ti_3_C_2_, etc.) [8], and hexagonal layered materials (h-BN) [9]. Despite sharing the common feature of reduced dimensionality, these materials exhibit markedly different electronic, optical, and thermal properties due to their distinct elemental compositions and crystal structures. Hexagonal boron nitride (h-BN) is an electrical insulator with a very high electrical resistivity (529 MΩ·cm) [10], exceptional thermal conductivity (751 W/mK for a monolayer at room temperature [11]), and a high Young’s modulus of 880 GPa [12]. In contrast, titanium carbide (Ti_3_C_2_) is metallic and highly conductive (2.4 MS/m [13]) while also being optically transparent, transmitting more than 97% of visible light [14]. These characteristics make it suitable for transparent conductive coatings and energy storage devices. Molybdenum disulfide (MoS_2_) is a semiconductor, which undergoes a transition from an indirect to a direct band gap when thinned from its bulk form to a monolayer [15], enabling unique optoelectronic behavior. Crystallographic parameters and band-gap values for these materials are summarized in Table 1 and Table 2, respectively.

**Figure 1:**
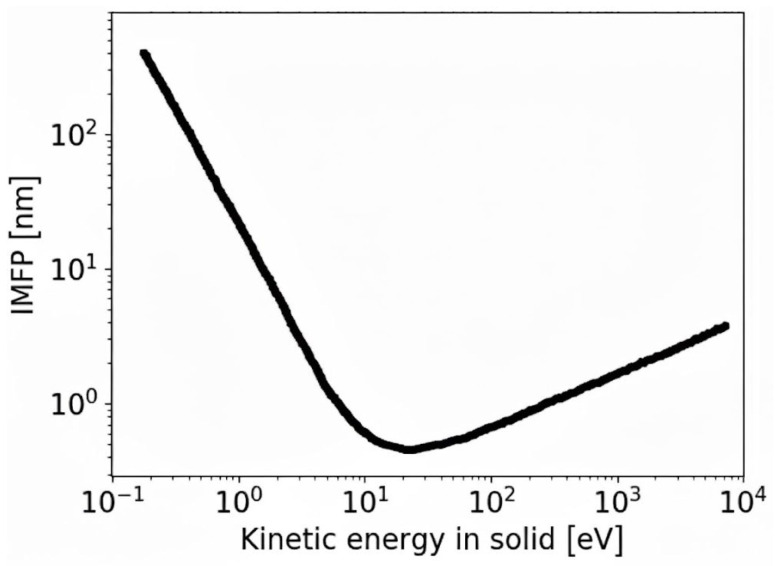
Universal curve of IMFP versus kinetic energy derived for amorphous materials [27,28].

New and largely unexplored 2D materials, together with their unique physical properties, require advanced analytical techniques for their comprehensive characterization. In particular, understanding electron–matter interactions in these systems is essential, making electron-based experimental methods a natural and powerful choice. A key physical parameter governing electron transport in solids is the inelastic mean free path (IMFP), defined as the average distance an electron travels between two inelastic scattering events. The IMFP strongly depends on the electron kinetic energy of electrons, a dependence commonly summarized by the so-called universal curve, illustrated in Figure 1. While this concept has been established for bulk materials, its validity for atomically thin 2D systems—where dimensionality and scattering mechanisms are fundamentally altered—remains an open question.

Measuring electron energy-loss spectra (EELS) provides direct access to the energy-loss processes governing electron transport and enables the determination of the IMFP. In this work, we employ an experimental approach that allows the acquisition of EELS from very slow electrons transmitted through ultrathin films. This methodology provides detailed insight into IMFP values and the underlying energy-loss mechanisms in 2D materials. By applying this approach to a range of 2D crystals, electron scattering phenomena—including surface excitations and layer-dependent effects—can be systematically investigated.

In this study, monolayer graphene was synthesized in-house via chemical vapor deposition (CVD), whereas h-BN, MoS_2_, and Ti_3_C_2_ were obtained commercially, and few-layer samples were produced through liquid-phase exfoliation (LPE). Subsequently, they were characterized using a combination of complementary techniques, including scanning low-energy electron microscopy (SLEEM), scanning transmission electron microscopy (STEM), Raman spectroscopy, and X-ray photoelectron spectroscopy (XPS). Building on our previous studies of graphene using an ultra-high-vacuum scanning low-energy electron microscope equipped with a time-of-flight spectrometer (UHV SLEEM/ToF) [29], we extend this approach to a broader set of 2D materials. Although the IMFP was calculated only for monolayer graphene at landing energies between 10 and 50 eV, it was not determined for the other 2D materials due to the unknown sample thickness. Nevertheless, the measured electron energy-loss spectra provide the essential experimental input from which the IMFP can, in principle, be derived, demonstrating the capability of low-energy EELS to probe electron scattering in 2D materials. Through this multifaceted approach, we aim to shed light on the unique potential of these 2D materials and their suitability for diverse technological applications.

## 2. Materials and Methods

### 2.1. Materials Preparation

Except for graphene, all 2D materials used in this study—h-BN, MoS_2_, and Ti_3_C_2_—were commercially sourced and subsequently used for the preparation of 2D samples. h-BN was purchased from Saint-Gobain, Amherst, NY, USA. Ti_3_C_2_ MXene powder was obtained from 2D Semiconductors, Scottsdale, AZ, USA. A pristine nanoflake solution of MoS_2_ was purchased from Graphene Supermarket, Calverton, NY, USA.

Materials were prepared according to the requirements of each characterization technique. The as-purchased MoS_2_ solution was briefly sonicated to ensure homogeneous flake dispersion prior to use. The Ti_3_C_2_ (MXene) and h-BN powders were exfoliated via LPE. While both were dispersed in isopropyl alcohol (IPA) the MXene solution was sonicated for 1 h, while h-BN solution was stirred for 5 min. Each dispersion was centrifuged at 500 rpm for 30 min to isolate the supernatant containing few-layer nanosheets. CVD graphene used in this study was grown in a custom-built reactor on copper foils (25 μm, ≥99.8%, MTI Corporation, Richmond, CA, USA) at 1050 °C and 1000 Pa. Using a modification of a standard method [30], precursor flows were set to 10 sccm H_2_ and 30 sccm CH_4_. Graphene was transferred onto substrates via a polymer-assisted wet transfer method, where a poly(methyl methacrylate) (PMMA) support layer (950k, 4% in anisole) was spin-coated onto the sample, followed by copper etching in FeCl_3_ and final PMMA removal in acetone.

Different substrates were used according to the requirements of each characterization technique. For SLEEM and Raman spectroscopy characterization, the resulting dispersions of MoS_2_, Ti_3_C_2_, and h-BN were drop-cast multiple times onto silicon substrates to obtain bulk samples, while CVD-grown graphene was transferred onto the silicon substrate. For STEM and SLEEM/ToF measurements, the dispersions were drop-cast once onto lacey carbon copper grids (300 square mesh, Agar Scientific, Sheffield, UK) and graphene was transferred onto a transmission electron microscopy (TEM) grid. Samples prepared from solutions were heated at 200 °C to remove residual solvent. For XPS analysis, sample preparation differed. The MoS_2_ dispersion was drop-cast onto a freshly cut carbon substrate, heated at 200 °C to remove residual solvent, and mounted onto the sample holder using a piece of copper tape. On the other hand, Ti_3_C_2_ and h-BN powders were directly pressed into shallow basins in the sample holder, forming flat and thick layers. Finally graphene was transferred to a Pt disk with a 100 μm hole.

### 2.2. STEM and SLEEM Measurements

Sample observations were performed using SLEEM and STEM modes on a Magellan 400 L scanning electron microscope (Thermo Fisher Scientific, Waltham, MA, USA) equipped with a beam deceleration mode. This configuration enables independent control of the primary beam energy within the electron optics and the effective landing energy of electrons at the sample surface, allowing high spatial resolution to be maintained even at very low landing energies [31].

Low electron landing energies, close to the so-called critical energy of secondary electron emission (where the total emission yield approaches unity), were employed to effectively suppress electrostatic charging of the samples. This approach is particularly advantageous for imaging electrically insulating or beam-sensitive materials without the need for conductive coating. In addition, the low electron energies reduce radiation-induced damage and enhance surface sensitivity.

In the STEM mode, imaging was carried out using a segmented semiconductor detector for transmitted electrons, enabling efficient collection of transmitted signals and discrimination of different scattering angles. This detector configuration provides contrast sensitivity to local variations in sample thickness, density, and structure, while preserving the benefits of low-energy electron imaging, namely reduced beam damage and charging suppression.

The combination of SLEEM/STEM operation, beam deceleration technology, and a segmented STEM detector enabled stable, high-contrast imaging under conditions that would otherwise lead to significant charging or sample degradation at conventional accelerating voltages.

### 2.3. Monte Carlo Simulations

Monte Carlo simulations of electron–matter interactions were performed using the CASINO^®^ software (v2.5.1.0.) [32] to illustrate electron penetration under the applied conditions. For h-BN, a density of 2.1 g·cm^−3^ and a stoichiometric composition of B (50%) and N (50%) were assumed, with a simulation energy of 500 eV, monolayer thickness of 0.300 nm, and interlayer spacing of 0.333 nm. MoS_2_ simulations were performed using a density of 5.06 g·cm^−3^, an electron energy of 500 eV, a monolayer thickness of 0.301 nm, and an interlayer distance of 0.314 nm. For Ti_3_C_2_ MXene, a density of 4.2 g·cm^−3^ was used, with simulations performed at electron energies of 250 eV and 500 eV, assuming a monolayer thickness of approximately 1.2 nm and an interlayer spacing in the range of 1.1–1.3 nm.

### 2.4. Raman Measurements

All Raman measurements were conducted on a Raman spectrometer (inVia, Renishaw, Wotton-under-Edge, UK ). The experiments were performed using a 532 nm laser operated at 100% power, corresponding to approximately 4 mW at the sample surface. The laser was focused to a spot diameter of ~800 nm. An objective with 50× magnification was used to focus the laser beam on the sample with a spot diameter of ~800 nm. Five different locations of each sample were measured by 10× accumulated 1 s expositions. The obtained spectra were corrected for background in Wire™ software (v5.6). Afterwards, the spectra were analyzed in Fityk software (v1.3.1) [33].

### 2.5. XPS Measurements

XPS measurements were performed using the AXIS Supra photoelectron spectrometer (Kratos Analytical, Manchester, UK). The monochromatized Al Kα with emission current of 10 mA (150 W) was used for all measurements. The analyzed spots were approximately 1 mm^2^ for standard XPS but reduced to 30 μm^2^ for μ-beam XPS applied in the case of free-standing graphene. The charging was compensated by low-energy electrons (flood gun). The spectra were acquired in the normal emission and 54° incident geometry (the take-off angle is 0° while the incident beam angle is 54° relative to the surface normal). The high-resolution core level spectra were measured with a pass energy of 20 eV and step size of 0.1 eV. The free-standing graphene was located by the XPS-imaging tuned at the Pt 4f_7/2_ energy (zero Pt 4f intensity signifies a hole in the Pt substrate with the free-standing graphene). The graphene sample was measured with a microscopic, 15 μm X-ray beam (a total exposed area on a sample was ~30–45 μm^2^) at a hole (gr-C) and at the outside of the hole (gr-C/Pt), whereas other samples were measured with a macroscopic, ~1 mm^2^ X-ray beam. The total energy resolution, determined from the full width at half maximum (FWHM) of the Ag 3d_5_/_2_ peak measured at a clean Ag foil with a pass energy of 20 eV, was 0.5 eV.

High-resolution B 1s, N 1s, Ti 2p, C 1s, Mo 3d, S 2p and O 1s core-level photoelectron spectra were analyzed in the ESCApe software (version 1.6.1.1234). The binding energy scale was calibrated to 284.8 eV of the C-C component in the C 1s region [34]. The spectra were fitted by peaks of a Gaussian*Lorentzian profile with a blend of 0.3 and the Shirley background subtracted. The B-N, N-B, Ti-C and C-Ti components in the B 1s, N 1s, Ti 2p and C 1s regions, respectively, were fitted as asymmetric peaks [35,36]. The peak intensities were regarded as free parameters except for constraining the relative intensity ratio of spin–orbit doublets to 2:1 and 3:2 for 2p and 3d core level spectra, respectively. The absolute peak positions were fitted as free parameters, but the relative peak positions were constrained by fixing the binding energy components separation (such as spin–orbit doublet splitting). The atomic concentrations of elements were calculated using the relative sensitivity factors in the ESCApe built-in library.

### 2.6. SLEEM/ToF Measurements and IMFP

**Figure 2:**
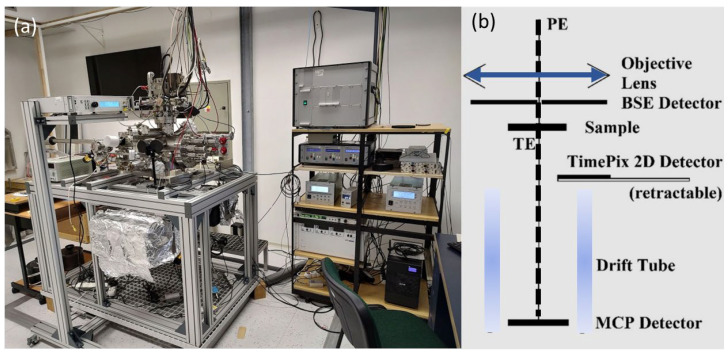
(**a**) UHV SLEEM/ToF experimental setup for ToF measurements under ultra-high vacuum conditions at the ISI CAS Brno. (**b**) Schematic diagram of the experimental apparatus.

For the analysis of 2D materials, the UHV SLEEM/ToF system [29] developed at the Institute of Scientific Instruments of the Czech Academy of Sciences (ISI CAS) in Brno was employed (Figure 2). The system integrates SLEEM with a ToF spectrometer for the detection of low-energy transmitted electrons. The microscope is equipped with a 5 keV primary Schottky electron gun from Delong Instruments Inc., Brno, Czech Republic [37], with an energy spread of 0.6 eV, and an in-house-build specimen stage with a biased holder enabling operation in cathode-lens (CL) mode [38]. This CL system allows an arbitrary low landing energy of electrons while preserving image quality and resolution [39]. The primary electrons in the beam can be decelerated by the strong electrostatic field generated in the CL mode to landing energies of a few eV. The ToF spectrometer consists of a drift tube, two focusing electrodes, and a multi-channel plate (MCP) detector, all enclosed within electromagnetic shielding. The momentum transfer of the aperture is 0.3 Å^−1^. The ToF spectrometer time resolution is 1 ns FWHM, while the energy resolution depends on multiple experimental parameters [40]. For a simulated flight time of 150 ns, the energy resolution is 0.5 eV and 0.17 eV for landing energies of 50 eV and 25 eV, respectively. However, the limiting factor for the overall energy resolution is the energy spread of the Schottky gun (0.6 eV). This demonstrates that the energy resolution improves at lower landing energies and that the energy resolution of a spectrometer is primarily determined by the energy resolution of the electron gun.

The system has custom MATLAB (vR2019a)- and C-based tracing scripts used for system control, optimization, energy calibration, energy conversion, and detector-image masking, enabling data analysis from a user-defined region of the MCP image after acquisition. The software enables optimization of the voltages applied to individual electrodes and selection of the *E_L_*. For each specific configuration, the software calculates the theoretical time-of-flight dependence *t*(*E*) (i.e., the time of flight as a function of electron energy for that configuration). This theoretical dependence serves as a conversion table between the time and energy domains and is applied automatically for each measurement [29,39]. The energy calibration is performed using the zero-loss peak (ZLP), defined as the time of flight corresponding to electrons that traverse the sample without energy loss. The ZLP is monitored in real time, and any systematic shifts are corrected by applying a linear time shift to the ToF timestamps [29,40]. This step ensures that the ZLP remains stable throughout the measurement and that its position corresponds to *E_L_* on the electron energy scale and to 0 eV on the electron energy-loss scale.

This unique system configuration enables the measurement of ToF spectra of transmitted electrons impacting the sample with energies on the order of a few eV, which is well suited for investigating electron interactions in 2D materials. While low-energy scanning electron microscopes (SEMs) typically operate in the range from hundreds of eV to a few keV, the present setup allows operation from a few eV up to several hundred eV, enabling ultra-low-energy ToF spectroscopy.

Measurements of the 2D materials were performed in pulsed mode using rectangular pulses with a 1.5 ns pulse width. The ToF spectra of electrons transmitted through the sample were recorded as timestamps of individual electrons. The measurement time for each sample was set to 30 min, measuring a spot of size on the order of nanometers. From the measured EEL spectra, the positions of the π and π + σ plasmon peaks were determined using the nonlinear curve-fitting software Fityk (v1.3.1) [33]. Individual plasmon features were fitted using Gaussian functions serving as models. The Levenberg–Marquardt (Lev–Mar) method implemented in Fityk was used for the fitting procedure. For MoS_2_ spectra containing multiple overlapping features, each plasmon contribution was fitted with an individual Gaussian component.

The IMFP of monolayer graphene was determined from the measured EELS following the data-processing procedure described in detail in Ref. [29]. Briefly, the acquired EELS were represented on a semilogarithmic scale (Y-axis). They were normalized to the intensity of the ZLP, which was subsequently fitted using a Gaussian function, which served as a model for the elastic peak. The IMFP was then extracted using the log-ratio method [41], yielding

(1) $$\lambda_{IMFP}(E_{L})=\frac{d}{ln\frac{S_{total}}{S_{ZLP}}}$$

where *E_L_* is the landing energy, *d* is the sample thickness, and *S*_ZLP_ corresponds to the integrated intensity of the fitted Gaussian model of the zero-loss peak, while *S*_total_ represents the integrated intensity of the entire energy-loss spectrum, excluding the secondary-electron (SE) peak at the end of the measured EELS. The resulting IMFP values depend on the experimental collection geometry and the detector acceptance, which determine the range of momentum transfer contributing to the measured spectra.

## 3. Results

### 3.1. STEM and SLEEM Measurements

**Figure 3:**
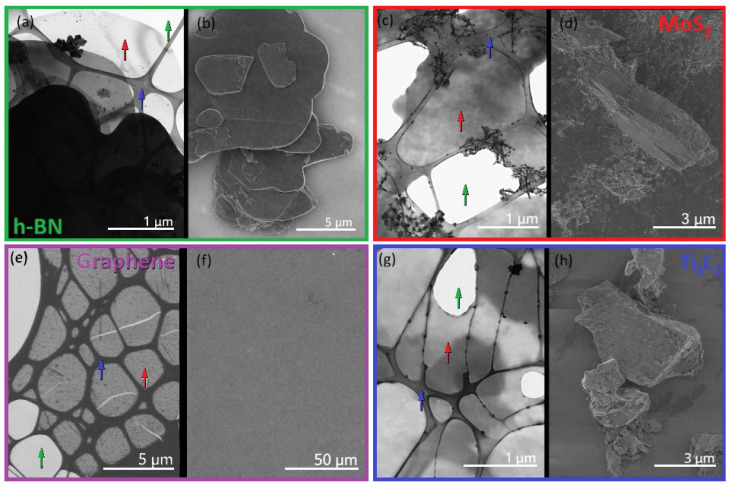
Comparative electron microscopy characterization of selected 2D materials using STEM and SLEEM. STEM images (**a**,**c**,**e**,**g**) were acquired from samples on a lacey carbon copper TEM grid, whereas SLEEM images (**b**,**d**,**f**,**h**) were obtained from samples on a silicon substrate. Red arrows indicate 2D layers/flakes (graphene, h-BN, MoS_2_, Ti_3_C_2_ MXene), blue arrows mark the supporting TEM carbon mesh, and green arrows denote holes corresponding to suspended areas. (**a**,**b**) h-BN: (**a**) STEM image acquired at an electron energy of 20 keV, revealing suspended thin membranes and local thickness variations; (**b**) SLEEM image acquired at 0.5 keV, highlighting surface morphology and edge contrast with enhanced surface sensitivity. (**c**,**d**) MoS_2_: (**c**) STEM image acquired at 20 keV showing a porous network of thin MoS_2_ flakes; (**d**) SLEEM image acquired at 0.5 keV emphasizing surface texture and flake morphology. (**e**,**f**) Graphene: (**e**) STEM image acquired at 2 keV displaying crystalline graphene domains with internal contrast variations; (**f**) SLEEM image acquired at 0.5 keV showing a large-area, laterally homogeneous graphene surface. (**g**,**h**) Ti_3_C_2_ MXene: (**g**) STEM image acquired at 20 keV revealing stacked and delaminated Ti_3_C_2_ flakes; (**h**) SLEEM image acquired at 0.5 keV providing detailed surface-sensitive contrast of individual MXene flakes.

The combination of STEM and SLEEM allows complementary access to bulk-sensitive transmission contrast and surface-sensitive imaging, enabling comprehensive structural characterization of ultrathin 2D systems (Figure 3).

STEM operates at landing electron energies in the range of 0–30 keV. This enables high sensitivity to local thickness, mass density, and crystallinity, while significantly reducing beam-induced damage. These features are critical for the study of atomically thin and beam-sensitive 2D structures. The transmitted electron signal enables direct visualization of suspended membranes, thickness variations, and defects within individual layers [42].

SLEEM employs electrons with energies in the sub-keV range and offers extremely high surface sensitivity due to the limited penetration depth of low-energy electrons. This makes SLEEM particularly suitable for probing surface morphology, layer continuity, and edge structures of 2D materials while minimizing subsurface contributions.

Figure 3 presents a comparative STEM and SLEEM characterization of selected 2D materials, demonstrating the complementary sensitivity of the two techniques to bulk and surface properties. STEM measurements were performed on crystalline samples, which were subsequently directly transferred into the microscope for ToF spectroscopy used to acquire EELS, enabling direct correlation between structural and electronic information. SLEEM imaging at very low electron energies provides enhanced surface-sensitive contrast. All investigated samples were transparent to 500 eV electrons, which is essential for the combined SLEEM imaging and ToF-EELS analysis within a single experimental platform.

### 3.2. Monte Carlo Simulations

**Figure 4:**
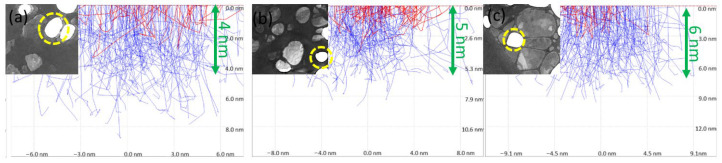
STEM imaging and Monte Carlo simulations illustrating the thickness requirements of two-dimensional materials for the ToF analysis at low impact energies. (**a**) Ti_3_C_2_ MXene, (**b**) MoS_2_, and (**c**) h-BN imaged by STEM at an electron energy of 0.5 keV. Contrast variations primarily originate from differences in local thickness and mass density, demonstrating the necessity of atomically thin regions for reliable ToF analysis at low impact energies. (The hole in the sample is highlighted in yellow in the STEM images. Red lines indicate primary electron trajectories, while blue lines indicate backscattered electrons).

From the measurements in STEM, we determined the lowest electron energy at which the flakes are still transparent. These electron energies were used in Monte Carlo simulations to establish the maximum thickness of individual samples with flakes on lacey carbon copper mesh, which were subsequently used in SLEEM/ToF measurement. These simulations emphasize the strong limitation of electron penetration depth at low energies and underline the requirement for ultrathin specimens in ToF-based surface-sensitive analyses (Figure 4). According to the Monte Carlo simulations, the samples must be only a few nanometers thick at most to allow sufficient electron transmission under these conditions. The transparency of graphene for low-energy electrons in the range of a few electronvolts has already been experimentally investigated and reported by the authors in previous studies, demonstrating the feasibility of electron transmission measurements at very low primary energies [43].

### 3.3. Raman Measurements

Raman analysis of all 2D samples was performed under identical conditions on the bulk materials, with the measurements performed on samples prepared on a silicon substrate. The silicon substrate produces a characteristic peak around 520 cm^−1^ [44] in the Raman spectrum, which does not overlap with the 2D material peaks. To account for the substrate contribution, a Raman spectrum of a clean silicon substrate was recorded and subtracted from the sample spectra. All resulting spectra were subsequently normalized for consistent comparison.

The Raman spectrum of graphene is characterized by three primary features: the 2D peak (≈2690 cm^−1^), the G peak (≈1580 cm^−1^), and the D peak (≈1350 cm^−1^) when using a 532 nm excitation source [45]. The G peak corresponds to a primary in-plane vibrational mode common to all graphitic structures, while the 2D peak represents the second-order overtone of a different in-plane vibration. The D peak, indicative of disorder in the crystal lattice, is typically absent in pristine graphene. The intensity ratio of the 2D to G peaks (I_2D_/I_G_) serves as a metric for determining layer thickness [45]. Identification of the number of graphene layers relies on a combination of the FWHM of the 2D band and peak intensity ratio I_2D_/I_G_. Generally, a ratio of ≈2 indicates a monolayer, ≈1 suggests a bilayer, and <1 signifies few- or multi-layer graphene. The FWHM for an ideal graphene monolayer is approximately 24 cm^−1^ [46], while for a bilayer, it is around 46 cm^−1^ [47].

**Figure 5:**
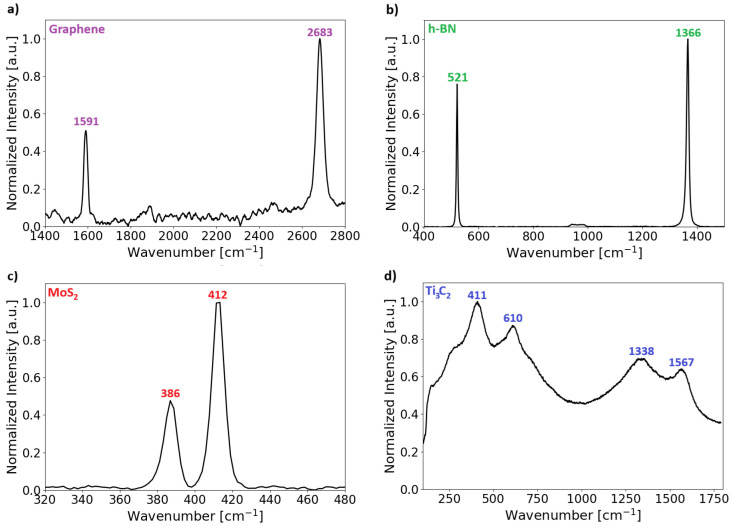
Raman spectroscopy characterization of the 2D materials measured on silicon substrates using a 532 nm laser. (**a**) Spectrum of CVD-grown graphene showing the characteristic G peak at 1591 cm^−1^ and the 2D peak at 2683 cm^−1^. The intensity ratio of the 2D to G peaks corresponds to single-layer graphene. (**b**) Spectrum of h-BN with characteristic peak at 1366 cm^−1^. (**c**) Spectrum of MoS_2_ with two specific peaks at 386 and 412 cm^−1^. (**d**) Spectrum of Ti_3_C_2_ with multiple characteristic peaks.

As shown in Figure 5a, signal noise obscures the detection of a potential low-intensity D peak. After normalizing the spectra, the I_2D_/I_G_ ratio was calculated to be ≈2, suggesting that the graphene on the substrate should be single-layered. The FWHM of the 2D peak is 41 cm^−1^, which is on the border of monolayer and bilayer graphene. In our case, the broadening is most likely caused by local strain, or small areas of bilayer graphene which, given a spot diameter of around 800 nm, averages the signal over this area. The ratio of the 2D and G peaks clearly indicates a monolayer, and for an ideal bilayer, the FWHM should be higher, so it is likely that this is a mostly monolayer graphene.

The Raman spectrum of h-BN is typically characterized by a dominant feature in the 1365–1367 cm^−1^ range, originating from the E_2g_ in-plane vibrational phonon mode [48]. As shown in Figure 5b, the measured spectrum exhibits a distinct h-BN peak at 1366 cm^−1^, accompanied by the characteristic silicon substrate signal at 521 cm^−1^. The number of h-BN layers can also be determined using the intensity of the main peak. The high intensity of the h-BN peak indicates the presence of a multilayered structure at the measurement point. However, to calculate the exact number of layers would require careful instrument calibration for a sample on the same substrate. Therefore, we will not attempt to determine the number of layers based on the main h-BN peak intensity here.

To assess crystalline quality, the FWHM was also analyzed. While polycrystalline or defective CVD h-BN typically displays broad peaks (>15 cm^−1^) [49], high-quality single crystals are defined by an FWHM of 8–10 cm^−1^ [50]. Our sample exhibits a narrow FWHM of 8.8 cm^−1^, confirming the material is a high-quality single crystal.

Figure 5c displays the Raman spectrum of the MoS_2_ sample. The number of layers was determined based on the frequency difference between the characteristic E^1^_2g_ (≈384 cm^−1^) and A_1g_ (≈405 cm^−1^) peaks [6]. It is well established that as the layer count increases, the A_1g_ peak blue-shifts due to stiffening interlayer van der Waals forces, while the E^1^_2g_ peak red-shifts. Consequently, a peak separation (Δ) of <20 cm^−1^ indicates a monolayer, whereas a separation approaching 25 cm^−1^ signifies bulk material. In our spectrum, the observed difference is 26 cm^−1^, confirming the presence of bulk MoS_2_. This result is expected, as the measurement points were specifically selected from regions with visible coverage under an optical microscope, which typically corresponds to thicker bulk material.

Figure 5d displays the Raman spectrum of the Ti_3_C_2_T_x_ MXene, which exhibits several distinct peaks in the range of 150–1600 cm^−1^. Unlike graphene or MoS_2_, the Raman signature of MXenes is complex as it depends heavily on the surface terminations (T_x_: -OH, -F, -O) and the stacking order. In the low-frequency region, the peaks observed at 411 cm^−1^ and 610 cm^−1^ are attributed to the E_g_ and A_1g_ vibrational modes of the MXene lattice, involving the motion of surface groups and titanium atoms [51]. Specifically, the shift in the peak to 609.97 cm^−1^ suggests the presence of -OH terminations [52], although minor peak shifts may also result from variations in the laser excitation wavelength (e.g., 514 nm vs. 532 nm).

Additionally, prominent peaks are visible in the high-frequency region at 1338 cm^−1^ and 1567 cm^−1^, corresponding to the D (disorder) and G (graphitic) bands of carbon, respectively. Both the G and D peaks are attributed to sp^2^ sites [53]. The occurrence of a G-band is linked to stretched C–C bonds in carbonaceous substances and is commonly associated with sp^2^ rings and chain architectures [54]. In contrast, the D peak occurs only in perturbed sp^2^ rings [55]. These findings align with expectations for Ti_3_C_2_T_x_, confirming that our sample contains a significant amount of free carbon (amorphous or graphitic), while the characteristic low-frequency peaks verify that the material is indeed Ti_3_C_2_T_x_ MXene.

### 3.4. XPS Measurements

The chemical composition of 2D materials characterized by means of XPS is shown in Table 3. Apart from oxygen and carbon, which are typical contaminants at surfaces exposed to the atmosphere [34,56] or amorphous carbon mesh, h-BN contains traces of silicon and sodium, while MoS_2_ contains traces of copper. The Si and Na contamination may be a result of processing or storing the sample in a liquid form in a glass vessel [57]. In the case of Ti_3_C_2_, there is a small concentration of nitrogen and a significant amount of aluminium incorporated in the sample. Aluminum probably originates from the initial Ti_3_AlC_2_ synthesis material [58].

**Figure 6:**
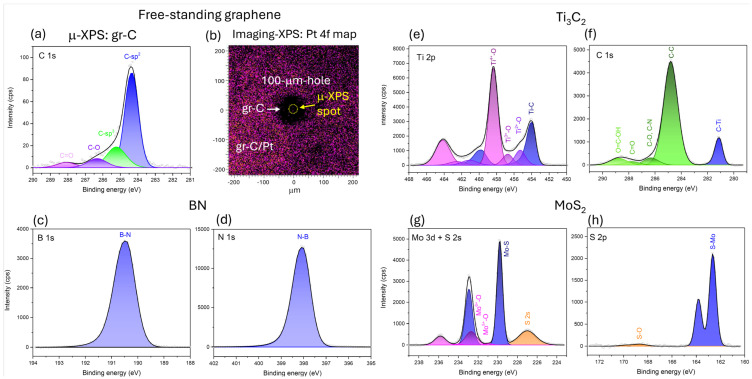
High-resolution XPS core level spectra of C 1s (**a**) from free-standing graphene, Imaging-XPS map of Pt 4f7/2 with graphene (**b**), B 1s (**c**), N 1s (**d**) from h-BN, Ti 2p (**e**), C 1s (**f**) from Ti_3_C_2_, Mo 3d (**g**), S 2p (**h**) from MoS_2_. All spectra are presented with the background removed.

Figure 6 presents XPS data acquired from (a,b) graphene, (c,d) h-BN, (e,f) Ti_3_C_2_, and (g,h) MoS_2_ samples. The C 1s spectrum of a free-standing graphene was measured over the hole region, as identified in the imaging XPS map in (b). The C 1s peak from graphene in (a) is dominated by contributions from sp^2^-hybridized carbon associated with the graphene layers; however, minor contributions from sp^3^-hybridized carbon and carbon–oxygen bonding configurations are also observed. The concentration of carbon–oxygen bonds is higher at the graphene/Pt region outside the hole, which is attributed to additional C–O contributions originating from the Pt support surface.

High-resolution core level XPS spectra of the h-BN contain only one component each, located at approximately 190.5 eV and 398.0 eV, which can be ascribed to boron nitride [59]. The atomic concentration ratio of B-N and N-B components, which is approximately 1.10, alongside the peak asymmetry of the B-N and N-B components, indicates the good quality of the h-BN 2D material [36,60].

The Ti 2p and C 1s core level XPS spectra of the Ti_3_C_2_ sample are shown in Figure 6e,f, respectively. The Ti 2p region consists of four 2p_3/2_–2p_1/2_ doublets with a split of 5.8 eV. The asymmetric Ti-C component at 454.0–459.8 eV can be assigned to titanium carbide [35]. The components at 458.4–464.2 eV, 456.7–462.5 eV and 455.3–461.1 eV originate from titanium oxides in different oxidation states: Ti^4+^-O, Ti^3+^-O and Ti^2+^-O, respectively [61,62]. The C-Ti component can also be seen in Figure 6f at 281.1 eV. Additionally, there are C-C (284.8 eV), C-O (286.3 eV), C=O (287.8 eV) and O=C-OH (288.8 eV) components from adsorbates and surface contaminants [34,56].

The MoS_2_ sample consists of the carbon substrate covered by a small amount of the 2D material (see Table 3). The core-level XPS spectra originating from the MoS_2_ are plotted in Figure 6g,h. The Mo 3d can be decomposed to three 3d_5/2_–3d_3/2_ spin–orbit doublets at 229.8–232.9 eV, 232.7–235.8 eV and 230.9–234.1 eV, and one broad S 2s peak at around 227 eV. The Mo 3d doublet at 229.8–232.9 eV corresponds to molybdenum sulfide (Mo-S) [63,64]. The Mo 3d components at 232.7–235.8 eV and 230.9–234.1 eV can be assigned to molybdenum oxides Mo^6+^-O and Mo^5+^-O, respectively [63,65,66]. Thus, Mo was partially oxidized.

The S 2p spectrum in Figure 6h is composed of a main 2p_3/2_–2p_1/2_ doublet at 162.6–163.8 eV, originating from molybdenum sulfide (S-Mo) [64], and a very small doublet at 168.6–169.8 eV, which can be attributed to sulfate [67]. The ratio between S-Mo/Mo-S components from the S 2p and Mo 3d regions is approximately 1.93, which is very close to the ideal MoS_2_ ratio of 2.00.

### 3.5. UHV SLEEM/ToF Measurements and IMFP

Measurements were performed at multiple spatially separated spots across the sample to assess reproducibility. However, for each landing energy, only a single spectrum was acquired per selected spot. A single acquisition per landing energy was chosen to minimize electron-beam-induced contamination and surface modification. MoS_2_, Ti_3_C_2_, and h-BN samples were prepared via LPE, while graphene was grown by CVD and subsequently transferred using a polymer-assisted wet transfer process based on PMMA. These widely used preparation methods may leave residual polymer contamination or other hydrocarbon adsorbates on the sample surface. In practice, a significant fraction of the hydrocarbon contamination observed during electron-beam experiments typically originates from residues already present on the transferred sample. Although the experiments were conducted under UHV conditions and the samples were thermally annealed prior to measurements to remove solvent residues and reduce surface contamination as much as possible, such effects cannot be completely eliminated. Repeated acquisitions over the same set of landing energies would therefore introduce systematic surface modifications rather than improve statistical precision.

**Figure 7:**
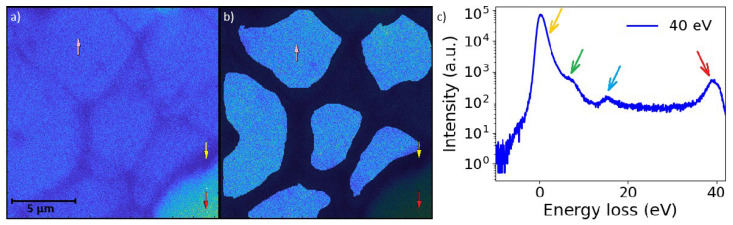
(**a**) MCP detector image of graphene on a lacey carbon copper mesh. The pink arrow indicates suspended graphene, the yellow arrow indicates lacey carbon, and the red arrow indicates a hole in the support structure. (**b**) Masked regions containing signal from suspended graphene used for spectral extraction. (**c**) Electron energy loss spectrum obtained from suspended graphene, showing characteristic features. The orange arrow indicates the ZLP, the green arrow marks the π plasmon, the blue arrow marks the π + σ plasmon, and the red arrow indicates the SE peak.

**Figure 8:**
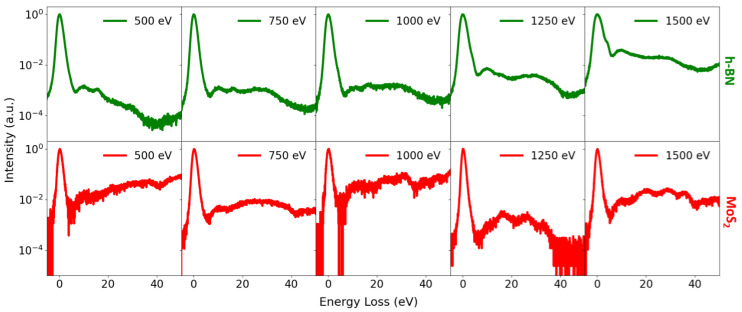
Measured EELS of h-BN (**top**) and MoS_2_ (**bottom**) acquired in the low landing energy range. For h-BN, distinct π and π + σ plasmon features are observed. In contrast, the MoS_2_ spectrum exhibits multiple loss peaks, indicating a more complex inelastic scattering behavior.

**Figure 9:**
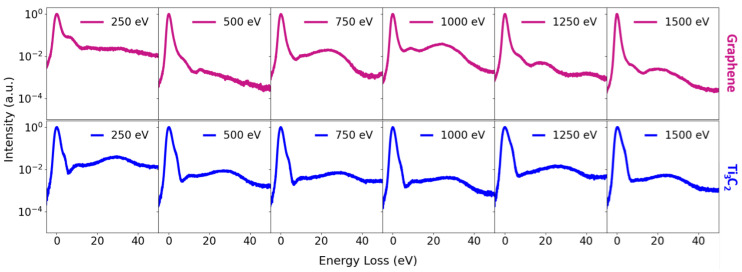
Measured EELS of monolayer graphene (**top**) and Ti_3_C_2_ MXene (**bottom**) acquired in the low landing energy range. In the obtained EELS, both π and π + σ plasmon peaks are clearly visible.

**Figure 10:**
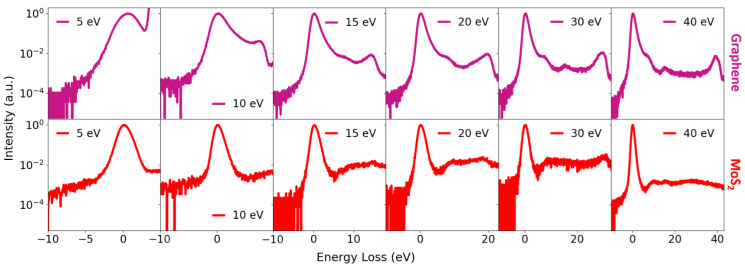
Measured low-energy EELS acquired in the very low landing energy range (5–40 eV). The graph shows spectra of graphene (**top**) and MoS_2_ (**bottom**). For graphene, the secondary-electron peak is observed, shifting toward higher loss energies with increasing landing energy. The MoS_2_ spectra exhibit multiple distinct loss features in addition to the ZLP, indicating complex low-energy inelastic scattering behavior.

The spatial distribution of the sample on the support mesh and the subsequent masking procedure used for spectral extraction are illustrated in Figure 7. ToF spectra were acquired for all investigated materials in the low landing energy interval between 500 and 1500 eV (Figure 8 and Figure 9). In addition, measurements in the very low landing energy range between 5 and 40 eV were performed for monolayer graphene and a few layers of MoS_2_, where sufficient transmission and signal-to-noise ratio could be achieved (Figure 10). The positions of the individual plasmon peaks were determined from the resulting EEL spectra and averaged over the range of landing energies. After acquisition, masking was applied to the MCP detector images to isolate signals originating from suspended flakes (Figure 7a,b). The measured EEL spectra can contain several characteristic peaks, depending on the landing energy of the electrons. The ZLP at 0 eV energy loss is always present, corresponding to electrons that did not lose energy during transmission. The π plasmon peak and the π + σ plasmon peak appear when the landing energy is sufficiently high—approximately 15 eV for the π plasmon and 30 eV for the π + σ plasmon. SE peaks are observed for landing energies below 50 eV (Figure 7c). Table 4 provides a comprehensive summary of the energy positions identified for the π and π+σ plasmon peaks, which were precisely determined from the EELS spectra acquired at various landing energies. This table is specifically designed to highlight the results obtained from the distinct set of samples investigated throughout our experiments, offering a direct and detailed comparison between these original experimental values and the corresponding data previously reported in the literature.

**Figure 11:**
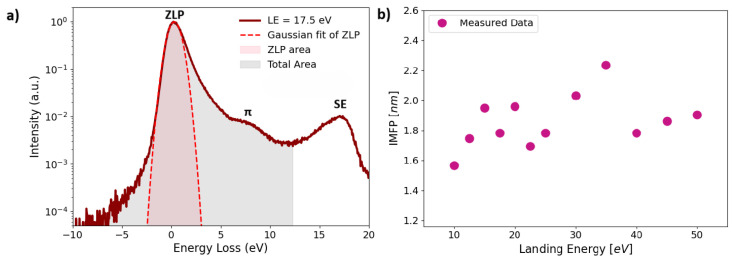
Determination of the IMFP for a monolayer of graphene in the very low landing energy regime. (**a**) EELS acquired at a landing energy of 17.5 eV, normalized to the ZLP, which is fitted using a Gaussian model (dashed line), and the shaded areas indicate the integrated ZLP and total spectral intensities used in the log-ratio method. The π plasmon and secondary-electron features are also indicated. (**b**) IMFP values of graphene extracted from the energy-loss spectra as a function of landing energy using the log-ratio method.

For graphene, the IMFP was determined in the very low landing energy interval using the log-ratio method applied to the processed EELS. The sample thickness (*d*) was taken as the theoretical monolayer thickness, estimated from bilayer graphene, with *d* = 3.35 Å [72]. The analysis involved normalization to the ZLP and Gaussian fitting of the ZLP, followed by integration of the fitted model as shown in Figure 11a. The resulting IMFP values are shown in Figure 11b. The average value of the IMFP in the electron landing energy range of 10–50 eV was found to be (1.9 ± 0.2) nm. For the remaining materials, IMFP determination was not possible due to the undefined sample thickness.

## 4. Discussion

In this work, we present a comprehensive characterization of representative two-dimensional (2D) materials—graphene, hexagonal boron nitride (h-BN), molybdenum disulfide (MoS_2_), and titanium carbide (Ti_3_C_2_) MXene—using a combination of structural, vibrational, chemical, and low-energy electron spectroscopic techniques. Particular emphasis was placed on electron energy-loss spectroscopy (EELS) performed using a unique home-built ultra-high-vacuum scanning low-energy electron microscope equipped with a time-of-flight spectrometer (UHV SLEEM/ToF) for transmitted slow electrons. Unlike conventional transmission electron microscopy (TEM) EELS, which typically operates at high electron energies, this approach enables measurements from a few eV up to the sub-keV range, providing enhanced surface and thickness sensitivity that is especially well suited for atomically thin and chemically heterogeneous systems.

The combination of scanning transmission electron microscopy (STEM) and SLEEM provides complementary structural information based on fundamentally different contrast mechanisms. While STEM relies on electron transmission and is sensitive to thickness, stacking, and crystallinity, SLEEM offers strong surface sensitivity governed by low-energy electron–matter interactions. Importantly, both techniques allow stable imaging of conductive and insulating materials without the need for conductive coatings, preserving beam-sensitive h-BN and chemically complex Ti_3_C_2_ MXene. All samples were prepared on TEM grids for STEM and on silicon substrates for SLEEM, enabling direct correlation between structural imaging and subsequent spectroscopic analysis.

Raman spectroscopy was employed to verify the structural quality and thickness of the investigated materials. Graphene exhibited a high I_2_D/IG ratio of approximately 2 and the absence of a pronounced D peak, confirming the high quality of the monolayer graphene. The Raman spectrum of h-BN showed a full width at half maximum (FWHM) of 8.8 cm^−1^, consistent with high-quality single crystals as reported previously. For MoS_2_, the separation between the E^1^_2_g and A_1_g modes (Δ ≈ 26 cm^−1^) confirmed that the material was bulk-like. The Raman spectrum of Ti_3_C_2_ MXene displayed characteristic low-frequency modes and a peak at ~610 cm^−1^ associated with –OH surface terminations, together with features attributable to free carbon, confirming the expected material composition and surface chemistry.

Low-energy ToF-EELS measurements were carried out under ultra-high-vacuum conditions to probe electronic excitations in all investigated materials.

For h-BN and MoS_2_, spectra were acquired in the 500–1500 eV landing energy range, whereas graphene and Ti_3_C_2_ MXene were measured over a broader interval from 250 to 1500 eV. In addition, very low landing energy measurements between 5 and 40 eV were successfully performed for graphene and MoS_2_. This restriction is dictated by the signal-to-noise ratio, as only these materials provided sufficient transmission and detectable signal in this energy regime. These results highlight the capability of the SLEEM/ToF approach to access interaction regimes that are not readily probed by conventional high-energy electron microscopy.

Across all measurements, the positions of the π and π + σ plasmon peaks were determined by fitting the corresponding loss features with Gaussian functions as a model using the nonlinear curve-fitting software Fityk [33]. The measured plasmon positions in our experiments differ from literature values, which is expected because the referenced studies employed different methods, including various experimental techniques and theoretical simulations. The plasmon energies are highly sensitive to experimental conditions such as collection geometry, momentum transfer *q*, instrumental resolution, and sample preparation method. The dependence of plasmon positions on momentum transfer has been demonstrated by simulations and EELS studies for graphene [29], h-BN [69], and MoS_2_ [73], where variations in scattering angle and collection conditions lead to measurable shifts in plasmon energies. In contrast, comparable simulations and low-energy EELS studies for Ti_3_C_2_T_x_ MXene remain limited. The present results therefore provide valuable experimental data and underline the need for further theoretical and experimental investigations of plasmon dispersion in MXenes.

For graphene, the inelastic mean free path (IMFP) was determined in the very low energy range of 10–50 eV using the log-ratio method applied to processed ToF-EELS. Accurate modeling and subtraction of the zero-loss peak is essential, as low-energy plasmons are also present in this region and have been recently discussed in [74]. A Gaussian function provided the best agreement with the experimental data. Our experimentally determined average IMFP is 1.9 ± 0.2 nm over a landing energy range of 10–50 eV. In contrast, Nguyen-Truong et al. [75] reported lower IMFP values in the same energy region based on time-dependent density functional theory (TDDFT) calculations, with averages of 1.01 ± 0.03 nm and 1.2 ± 0.7 nm for momentum transfer along the ΓK and ΓM crystallographic directions, respectively. This difference likely reflects the distinct nature of the approaches: the TDDFT results are direction-specific and capture anisotropic scattering along defined crystallographic paths, whereas the experimental values represent a direction-averaged IMFP, which can lead to systematically higher values. IMFP determination for the other materials was not feasible due to the unknown and non-uniform sample thickness.

In contrast to graphene, MoS_2_ and Ti_3_C_2_ MXene exhibited broader and less well-defined plasmon features, which can be attributed to their more complex surface chemistry. X-ray photoelectron spectroscopy (XPS) analysis revealed oxidation, mixed bonding states, and surface terminations that introduce additional inelastic scattering channels and enhance plasmon damping. These observations demonstrate the strong sensitivity of low-energy ToF-EELS to chemical heterogeneity and surface modifications, which may not be apparent from purely structural characterization.

Overall, this work establishes low-energy ToF-EELS in a home-built UHV SLEEM/ToF system as a powerful and complementary tool for probing electron–matter interactions in two-dimensional materials. When combined with STEM, SLEEM imaging, Raman spectroscopy, and XPS, this approach provides a comprehensive framework for studying surface-dominated electronic excitations, thickness-dependent scattering, and electron transport in ultrathin systems. The methodology is particularly promising for emerging 2D materials, functionalized layers, and heterostructures, where surface effects play a decisive role in determining physical properties. This perspective can be extended to new material systems, including intercalated compounds [76], in order to evaluate whether such an approach is also suitable and effective for their characterization.

## 5. Conclusions

This work demonstrates the capability of a home-built UHV SLEEM/ToF system equipped with time-of-flight spectroscopy to perform low-energy ToF-EELS on two-dimensional materials with high surface and thickness sensitivity. The combined use of SLEEM, STEM, Raman spectroscopy, and XPS enabled reliable structural, vibrational, and electronic characterization of graphene, h-BN, MoS_2_, and Ti_3_C_2_ MXene.

The presented low-energy ToF-EELS method combines the surface sensitivity of low-energy electron spectroscopy with the spatial resolution provided by low-energy SEM. In contrast to conventional EELS performed in transmission electron microscopes, which typically employ high-energy electron beams and probe excitations throughout the sample volume, the use of low-energy electrons enhances sensitivity to surface and near-surface electronic excitations while reducing beam-induced damage [77,78].

In conventional surface-science implementations such as high-resolution electron energy-loss spectroscopy (HREELS), measurements are typically performed in reflection (specular scattering) geometries and probe surface and near-surface excitations with high sensitivity [79]; however, such approaches generally probe relatively large surface areas and therefore do not provide intrinsic spatial resolution. In contrast, the present method combines a normally incident low-energy electron beam with SEM imaging, enabling spatially resolved measurements of electronic excitations. This configuration allows mapping of plasmonic modes, including π and π + σ plasmons, while retaining the strong surface sensitivity characteristic of low-energy electron spectroscopies [41].

A further important motivation for this method is its applicability to the determination of the IMFP at very low electron energies. Reliable experimental IMFP data in the few-eV to tens-of-eV range remain scarce, despite their critical importance for the quantitative interpretation of electron spectroscopies [80]. The normally incident geometry and well-defined low-energy electron beam make the present approach particularly suitable for systematic measurements required for IMFP determination in this poorly explored energy regime.

Future work will focus on improving energy resolution and applying the method to spatial mapping of plasmonic excitations in nanostructured and two-dimensional materials. In addition, systematic measurements on well-defined reference materials may provide new experimental data for low-energy IMFP determination, contributing to improved quantitative models for electron spectroscopy and surface analysis.

## Figures and Tables

**Table 1:** Crystallographic and structural parameters of selected 2D materials.

Material	Crystal Lattice	Lattice Parameters [Å]	Monolayer Thickness [Å]	Distance Between Layers [Å]	Ref.
Graphene	hexagonal	a = b = 2.46	3.5	3.35	[16,17]
h-BN	hexagonal	a = b = 2.50	4	3.33	[18,19,20]
Ti_3_C_2_	hexagonal	a = b = 3.05 c = 19.86	10	13.5	[21,22,23]
MoS_2_	hexagonal	a = b = 3.15 c = 12.3	6.5	6.2	[24,6,25]

**Table 2:** Band gap type and values for selected materials.

Material	Type of Band Gap	Band Gap [eV]	Ref.
Graphene	direct	0	[26]
h-BN	direct	5.9	[12]
Ti_3_C_2_	—	—	—
MoS_2_	direct	1.8	[12]
	indirect	1.2	[12]

**Table 3:** Atomic concentration of elements (at.%) derived from XPS quantitative analysis.

Sample	B	N	O	C	Ti	Mo	S	Si	Na	Al	Cu	Pt
gr-C/Pt	—	2.6	11.5	82.8	—	—	—	—	—	—	—	3.1
gr-C	—	—	15.7	84.3	—	—	—	—	—	—	—	—
h-BN	41.7	45.7	2.7	8.6	—	—	—	0.3	1.0	—	—	—
Ti_3_C_2_	—	1.8	40.3	35.4	13.1	—	—	—	—	9.4	—	—
MoS_2_	—	—	26.1	91.8	—	1.3	2.1	—	—	—	0.3	—

**Table 4:** Positions of the π and π + σ plasmon peaks obtained from EELS measured at different landing energies, compared with reference values reported in the literature for the corresponding materials.

Material	π [eV]		π + σ [eV]	
	Exp	Ref	Exp	Ref
Graphene	6.7 ± 1.1	4.6 [68]	19 ± 4	14.6 [68]
h-BN	10.0 ± 0.3	12.3 [69]	26.5 ± 1.5	30.3 [69]
Ti_3_C_2_	9.9 ± 0.8	10 [70]	26.6 ± 1.6	24 [70]
MoS_2_	10.3 ± 0.7	8.6 [71]	26.7 ± 1.7	23 [71]

## Data Availability

The original contributions presented in this study are included in the article. Further inquiries can be directed to the corresponding author.

## Abbreviations

The following abbreviations are used in this manuscript:

2D	Two-Dimensional
CL	Cathode Lens
CVD	Chemical Vapor Deposition
EELS	Electron Energy-Loss Spectra
FWHM	Full Width at Half Maximum
h-BN	Hexagonal Boron Nitride
HREELS	High-Resolution Electron Energy-Loss Spectroscopy
IMFP	Inelastic Mean Free Path
IPA	Isopropyl Alcohol
ISI CAS	Institute of Scientific Instruments of the Czech Academy of Sciences
LPE	Liquid Phase Exfoliation
MCP	Multi-Channel Plate
STEM	Scanning Transmission Electron Microscopy
MoS_2_	Molybdenum Disulfide
PMMA	Poly(Methyl Methacrylate)
SE	Secondary Electron
SEM	Scanning Electron Microscope
SLEEM	Scanning Low-Energy Electron Microscopy
TDDFT	Time-Dependent Density Functional Theory
TEM	Transmission Electron Microscopy
Ti_3_C_2_	Titanium Carbide
TMD	Transition Metal Dichalcogenide
ToF	Time of Flight
UHV	Ultra-High Vacuum
XPS	X-Ray Photoelectron Spectroscopy
ZLP	Zero Loss Peak
